# Enablers of psychosocial recovery in pediatric burns: perspectives from the children, parents and burn recovery support staff

**DOI:** 10.1186/s12887-020-02180-z

**Published:** 2020-06-09

**Authors:** Ashley Van Niekerk, Roxanne Jacobs, Nancy Hornsby, Robyn Singh-Adriaanse, Mathilde Sengoelge, Lucie Laflamme

**Affiliations:** 1 Violence, Injury and Peace Research Unit, South African Medical Research Council and University of South Africa, Tygerberg, South Africa; 2 Institute for Social and Health Sciences, University of South Africa, Johannesburg, South Africa; 3 The Alan J. Flisher Centre of Public Mental Health, University of Cape Town, Cape Town, South Africa; 4 Department of Public Health Sciences, Karolinska Institutet, Stockholm, Sweden

**Keywords:** Pediatric burn, Psychosocial recovery, Enablers, Children, Caregivers, Burn care providers

## Abstract

**Background:**

Pediatric burn injuries are a major cause of death and injury, occurring mainly in resource poor environments. Recovery from burns is widely reported to be constrained by physical, psychological, relationship and reintegration challenges. These challenges have been widely described, but not the enablers of psychosocial recovery. This is especially true in pediatric burn research, with few multi- perspective studies on the recovery process.

**Methods:**

This qualitative study involved 8 focus group discussions (four with 15 children post-burn injury, four with 15 caregivers) and 12 individual interviews with staff working in pediatric burns that explored the psychosocial needs of children after a burn and the enablers of their recovery. Purposive sampling was utilized and recruitment of all three categories of participants was done primarily through the only hospital burns unit in the Western Cape, South Africa. The interviews focused on factors that supported the child’s recovery and were sequentially facilitated from the child and the family’s experiences during hospitalization, to the return home to family and friends, followed by re-entry into school. Thematic analysis was used to analyze verbatim interview transcripts.

**Results:**

The recovery enablers that emerged included: (i) Presence and reassurance; indicating the comfort and practical help provided by family and close friends in the hospital and throughout the recovery process; (ii) Normalizing interactions and acceptance; where children were treated the same as before the injury to promote the acceptance of self and by others especially once the child returned home; and (iii) Sensitization of others and protection; signifying how persons around the child had assisted the children to deal with issues in the reintegration process including the re-entry to school.

**Conclusions:**

This study indicates that the psychosocial recovery process of children hospitalized for burns is enabled by the supportive relationships from family members, close friends and burn staff, present during hospitalization, the return home, and school re-entry. Support included comfort and physical presence of trusted others and emotional support; affirmation of the child’s identity and belonging despite appearance changes; and the advocacy and protection for the re-entry back into the school, and more generally the community.

## Background

Pediatric burns are a public health problem globally, concentrated in resource constrained regions where burns are highest in countries with low economic levels, such as in Sub-Saharan Africa [1]. A burn injury is considered one of the most traumatic, painful and stressful trauma experiences with disrupting effects on normal life [2–4] due to both the physical consequences of the injury and the stigma and discrimination associated with disability and in many instances, disfigurement [5]. Severe pediatric burns may lead to growth and development delays, behavioral and social problems within or outside the family [3], and schooling interruptions [6, 7]. Survivors can suffer serious short- and long-term consequences among which well-known psychological outcomes are post-traumatic stress disorder, depression, anxiety and sleep disturbances [7], increased aggressiveness, disturbed self-esteem and distressing memories of the burn [8]. These sequelae are likely exacerbated in adverse family and community settings, as is common in South Africa, where burn rates are high [1], where there is limited access to formal and specialized health, rehabilitation and support services [9], and where community stigmatization of survivors is a concern and may be widespread [10].

Studies on survivor perspectives as regards the prospects of recovery highlight how two main trauma phases come into play: the burn event (i.e. initial trauma of experiencing the burn) and the recovery itself. The latter encompasses a range of experiences within the child and between the child and the environment. These include: recurrent traumatization due to invasive medical procedures in wound management; scarring acting as a permanent reminder of the trauma even long after the wound has healed; bullying due to visible differences; behavioral changes such as avoidance, hyper- vigilance, and internalizing symptoms; and family reactions and adaptation in the form of overprotective parenting or child parentification [4].

Adequate and timely psychosocial^1^ support to victims is essential in order to maximize physical and psychological recovery and reintegration back into the home, broader community and school [11]. The delivery of effective clinical and post-trauma interventions, including pain management interventions and early treatment of post-traumatic stress, thus play a determinant role in mitigating the consequences of these injuries. An increasing body of knowledge however focuses beyond the impairments in the injured child and emphasizes factors that enable recovery in a strengths-based approach [12]. This approach has often referred to the concept of resilience, i.e. the “process of overcoming the negative effects of risk exposure, coping successfully with traumatic experiences, and avoiding the negative trajectories associated with risks” [13]. Resilience studies recognize risk exposure but are focused on strengths rather than deficits and on understanding the processes that inform healthy development despite adversity [13]. Such studies have highlighted the interplay between a child’s capacity to use opportunities, the capacity of the family and environment to provide resources to facilitate the child’s recovery, and the contextually relevant interactions in the recovery process [12, 14, 15]. This provides a comprehensive picture of enablers at various levels, rather than focusing only on the personal characteristics of the vulnerable child’s ability or inability to show resilience [16]. Innate enablers of recovery include a pragmatic attitude towards and acceptance of (the inevitability of) pain while progressing through rehabilitation, as well as acquiring hope and actively visualizing a future that includes set rehabilitation goals and a return to a previous or new vocation [17, 18]. External enablers include support from family, friends, peers and professionals for dealing with the trauma and providing positive, uplifting relationships, as well as being informed about medical procedures and included in decisions [17, 18], and being shielded from stigma [19]. The supportive individual and relational factors to recovery have been documented for the trauma experienced after other causes of acute injuries [20] including adult burns [21]. However there have been few studies that have focused on the factors that enable the recovery of children after burns (e.g. [12], with few if any describing enabling factors as these may be pertinent to the immediate to longer term phases of recovery. The exploration of such factors is important for guiding enabler focused interventions and well suited to qualitative study.

The current study is thus one of the first qualitative investigations on the enablers important to pediatric burn recovery and over key phases in its progression. It is one of the few that combines perspectives from the child, parents and burn unit staff who have had direct, often close contact with the children over considerable periods of time [22]. This study aims to clarify these enablers, and thus contribute to this emerging knowledge of the factors that support the recovery process of burn injured children, especially in adverse settings.

## Methods

### Setting

The study took place in the Western Cape Province, South Africa, which has an estimated 6.5 million inhabitants [23]. Pediatric burns are a considerable public health problem in the Province; both in prevalence [24], and often severity, with a mortality of 3.6/100000 person-years for those aged 15 years and younger in Cape Town, the provincial capital [25]. These mainly affect children from the poorest segments of the population and occur in and around homes with inadequate living conditions [25, 26].

### Design and participants recruitment

The study is qualitative and combines three sets of data all addressing similar questions but dealing with three different groups of participants for complementary perspectives [27]: children who suffered a burn injury, caregivers of these children and professionals, and mainly hospital burn unit staff but also volunteers and non-governmental organization (NGO) staff interacting with them. Inclusion criteria for the children was (i) age 10 to 15 years; (ii) hospitalized in the burn unit for at least one night; 3) discharged for at least six months but not more than three years prior to contact with the research group. Children of this age group were chosen so that they had the required cognitive capacities, emotional maturity, independence, and verbal skills for engaging in discussions on their recovery experiences with minimal re-traumatization [28–30]. Table 1 presents characteristics of the participants with the level of detail adjusted to protect the privacy of the participants.

**Table 1:** Participant characteristics

Target group	Type of interview	Occupation (if applicable)	Gender		Length (in minutes)
			Female	Male	
Children (9–15 yrs)	FGD 1		4	–	42
	FGD 2		4	1	39
	FGD 3		2	1	63
	FGD 4		2	1	28
Family	FGD 5		4	–	72
	FGD 6		5	–	46
	FGD 7		3	–	77
	FGD 8		3	–	75
Professionals: Burns unit	Interview 1	Therapist	1	–	35
	Interview 2	Therapist	1	–	84
	Interview 3	Volunteer	–	1	41
	Interview 4	Psychologist	1	–	53
	Interview 5	Nurse	1	–	68
	Interview 6	Social worker	1	–	76
	Interview 7	Volunteer	1	–	68
	Interview 8	Volunteer	–	1	36
	Interview 9	Therapist	1	–	28
	Interview 10	Nurse	1	–	32
	Interview 11	Nurse	1	–	60
	Interview 12	NGO staff	1	–	60

The three groups were recruited by means of purposive sampling through the Red Cross War Memorial Children’s Hospital Burn Unit, the only specialized pediatric burns unit in Africa with the vast majority of patients from under-resourced, low income communities. Caregivers of 42 children were called by members of the research team (RJ, NH) to assess eligibility based on the inclusion criteria. Two child focus group discussions (FGDs) and two caregiver FGDs were scheduled in this way. Snowballing techniques were then employed to obtain two more child FGDs and two caregiver FGDs via personal contacts with burn survivors in the local community. All caregivers and children who met the criteria and agreed to participate in the study were invited to attend an orientation session at the Red Cross Hospital which took place immediately before the FGDs, lasting between 10 to 15 min. These sessions sought to fully brief participants and once parents had consented for their children to provide consent themselves, children and parents were separated to obtain individual consent to ensure that children did not feel obligated to participate. The recruitment of professionals was supplemented with a web-based search of staff from burn survivor support non-governmental organizations in South Africa.

### Data collection

One interview guide to obtain feedback with the professionals and two facilitation guides, one for the child FGD and one for the parent FGD (see Additional file 1, 2 and 3) were consistently used throughout the interviews. The guides were designed prior to data collection and were based on a review of relevant literature combined with the research team’s extensive experience in child burn epidemiology and prevention. The focus of the interview guides was on the psychosocial issues and needs of children after a burn injury and the enablers for recovery. Items for the child group were piloted on 2 healthy, non-injured children aged 12 and 14 years and no changes made.

Data collection took place from July 2017 to May 2018 for 12 individual interviews with the professionals and eight FDGs with 30 participants (15 children, 15 caregivers). Two interviewer-facilitators (drawn from AVN, RSA, NH, RJ) were present for each session, conducted at venues convenient to the participants. Permission and informed consent to audio-record the interviews/FDGs were obtained for each session, and conducted in three languages: English, Afrikaans and isiXhosa. The length of the interviews and FGDs ranged from approximately 28 to 84 min. No changes were made to the interview guides with regards to the questions, which were firstly open ended and thereafter more specifically focused to enable the exploration of emerging issues. Therefore, varying and ‘new’ issues emerged with successive interviews/FGDs and this process was continued until we reached a consensus based the saturation of issues. The interviews were audio recorded, transcribed verbatim or intelligent verbatim and translated to English. Both interviewers checked the content of the transcriptions independently.

### Data analysis

Thematic analysis [31] was used to perform the data analysis. Data familiarization was performed by transcribing the data and reading and rereading the data, noting down initial ideas. Four of the authors from the research team (AVN, RSA, NH, RJ) performed data analysis with teams of two independently analyzing the interviews from the three informant groups, i.e. for the child (RJ, RSA); caregiver (RSA, NH) and health care provider interviews (NH, RJ), and an author (AVN) leading the verification process for each informant group. Interrater agreement was checked for initial codes via discussion between the coders in each team, with the full research team thereafter involved in the compilation of the sub-themes and themes. Themes were checked to ensure relevancy in relation to the codes. This process generated a ‘thematic map’ of the analysis. Then quotes from the participants were examined to portray the themes and sub-themes in the participants’ own words. Consensus building took place within the research team to finalize this inductive process of maintaining consistency between the data presented and the formation of the themes and sub-themes.

## Results

**Fig. 1:**
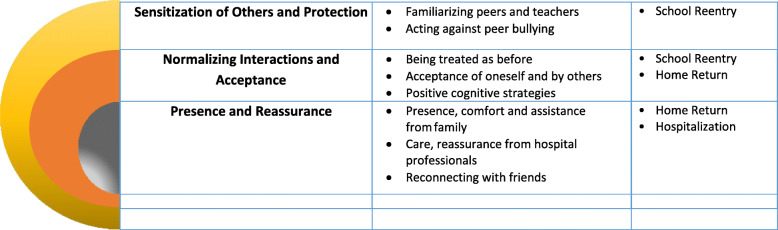
Enablers of psychosocial burn recovery that emerged as themes and sub-themes from the thematic analysis

Recovery of the child after a burn takes place through key experiences occurring at the hospital, in the return home and in reintegration back into the community. Three themes emerged from the thematic analysis: (i) Presence and reassurance; from family, friends and hospital staff relationships; (ii) Normalizing interactions and acceptance; referring to those interactions where the child is treated the same as before the injury to promote the acceptance of self and by others; and (iii) Sensitization of others and protection; signifying the educational efforts directed at persons around the child to enable their support of the child in dealing with issues in the reintegration process, and strengthen their protection of the child from traumatization, e.g. through bullying. Figure 1 provides a visual representation of the three themes and eight sub-themes, and the phases, within which these were primarily manifest. The child and the persons most influential at the start of the recovery process in the burn unit are depicted at the core of the circle in grey, followed by the orange circle representing the child returning to the home and everyday neighborhood environment, and the yellow circle representing the wider community, especially the school environment as the place where children spend the majority of their time.

### Theme 1: presence and reassurance

Theme 1 is comprised of three sub-themes, i.e. (i) Presence, comfort and assistance from family; (ii) Care and reassurance from hospital professionals; and (iii) Reconnecting with friends. Key illustrations of each is provided below. All the groups highlighted the Presence, comfort and assistance from family; while Care and reassurance from hospital professionals were mostly indicated by health experts followed by parents and children; and Reconnecting with friends by both caregivers and children.

#### Presence, comfort and assistance from family

The physical presence, emotional and practical support started with family visits in the hospital’s Burn Unit which comforted children and bolstered their feelings of being cared for, especially immediately after the burn. This physical presence was essential, especially from parents, and was complemented with reassuring communications and gestures that “things will be fine” (Parent, Interview 2, pg. 3). Furthermore, it was important for children to know that even if family could not physically be in the room, just the knowledge of their presence in the hospital facilitated both their physical and emotional recovery:

... Because my child, she was burned … and like the next day … most of my family came to see her. She wasn’t close with them, but that day, when she was burned … They came to see her and they weren’t allowed here but they were standing outside, so that they can see her and that, I promise you that … was helping her to heal herself in the hospital .... [Parent, Interview 1 pg. 9–10].

Supplementing this presence of family was the significance of practical everyday help which was pervasive and stood out in the children’s accounts of their experiences especially on their return home from hospital. Family members, particularly siblings, would e.g. take over the children’s chores, such as doing the dishes, on occasion the laundry, and preparing food. One child drew attention to his sister’s emotional support when he felt unwell and even helpless; another’s would be physically affectionate by hugging him and buying him treats; while others highlighted the resumption of play with siblings; some, the household chores done by siblings, including one by sibling who uses a wheel-chair; and another the physical escort provided by a sibling in guiding him around while his vision was still impaired by bandaging.

#### Care and reassurance from hospital professionals

Hospital care providers provided not only physical healing support but also through a nurturing role intentionally provided emotional support to the children and parents as they adjusted to the shock of the burns and the painful hospital procedures (e.g. initial burn dressing changes). They also allowed children to participate in decision-making around aspects of their medical care, helped with practical matters such as calls on behalf of the child to the mother, and reassured the child on the continuity of care even with staff rotations or departures. One child highlighted the role that the nurses and doctors had played as central to survival:

… When I left the hospital, then I thanked all the nurses and the doctors for helping me. And for when they carried me out … and for all the work they have done, it was very beautiful...If it wasn’t for them, I would not have made it. [Child 1, FGD 3, p.8].

This supportive role was supplemented by ongoing pain management identified by experts as underpinning the child’s ability to cope with burn wound care. Pain control alleviated emotional distress, strengthened the experience of coping, and offered the child some experience of control:

The other thing, we [were] looking at in pain management … so, one of the things we identified was giving the child back his control … where the child is allowed then to maybe take off his own dressing [Professional, Interview 5, pg. 6].

The children’s anxieties were lessened when hospital staff engaged with the children and took the time to speak to them directly. Hospital volunteers, particularly those who are burn survivors, were of particular assistance in engaging the child in the recovery process, specifically through reading and where possible play, thus providing comfort to the child but also serving as an example of survival and coping, and thus reducing anxiety.

#### Reconnecting with friends

When the children returned home they experienced stigmatization and mocking by others, but also the importance of friends in strengthening their ability to cope. The significance of both instrumental support and affection was highlighted by the children and experienced through a warm welcome back home, gift-giving and gestures of friendship, in a number of cases from friends made in hospital, but especially from those that had been regarded as best friends. The resumption of play activities was important, and the company when introduced back into the neighborhood. The significance of these friendships was also echoed by the parents who elaborated that although some children were initially resistant to reengaging with friends, maintaining friendships despite the occurrence of the burn was crucial:

… Her friends she always played with at home, those. Even, even, even if she says no, friends must come. Even if they come every day, even if they come every second day or whatever. But as long as she, she can see in her mind, her mind tells [and she] can see it: ‘No, they want to be my friends’. [Parent 3, FGD 1].

### Theme 2: normalizing interactions and acceptance

Theme 2 includes three sub-themes: (i) Being treated as before; (ii) Acceptance of oneself and by others; and (iii) Positive cognitive strategies, each of which is demonstrated below. Caregivers highlighted Being treated as before; while Acceptance of oneself and by others were mostly indicated by health experts; and Positive cognitive strategies by children followed by caregivers.

#### Being treated as before

The children relied on family and close friends to engage them to participate in everyday, social activities, such as holding the child’s hand in public, visiting the mall and places of entertainment, taking photographs of the child, and having sleepovers with friends:

… I would ask her to go with, avoiding her being lonely. We would take pictures at the mall avoiding the feeling of her thinking that I’m no longer loving her since she got burnt. …. I would make sure that I also style her hair up whenever I’m styling her other sister to avoid treating my children differently … I wanted her to see that to me she’s still the child that she was even if she got burnt. [Parent 1, Interview 4].

#### Acceptance of oneself and by others

The health workers affirmed the acceptance by others’ as an important step towards the child’s self- acceptance. In particular, one father had recognized and asserted his daughter’s value which, in turn, encouraged her self-acceptance and hope for a life after the burn:

I must say that her inner strength … that she found within herself … was due to the fact that her dad accepted and acknowledged his beautiful little girl and because he acknowledged it, she acknowledged it, that she is the little girl who is alive, that can still live and be out there … and have a life. [Health worker Interview 5].

Another child made a conscious effort to see himself in the mirror and channel his own resources inwardly to promote self-acceptance:

But with the boy that I referred to earlier, he then made a point to always go to the mirror and look at himself and really got better … he really did get better … [Health worker Interview 5].

#### Positive cognitive strategies

Also important were the children’s use of individual cognitive coping strategies to bolster the child and indirectly others’ acceptance of the changes brought about by the burn injury. These centered around positive self-talk around the child’s perseverance, maintaining physical strength, and managing their fears, for example:

You can just focus on one thing at a time. Not too much, like um … if you come out of hospital, just keep yourself relaxed. Don’t worry about what’s going to happen tomorrow. [Child 4, FGD 1].

### Theme 3: sensitization of others and protection

Theme 3 included two sub-themes: (i) Familiarizing peers and teachers; and (ii) Acting against bullying. Caregivers, but also experts and children highlighted the importance of Familiarizing peers and teachers; while mostly children and some caregivers foreground Acting against bullying.

#### Familiarizing peers and teachers

A supportive school environment in which the child feels a sense of belonging was identified as an important factor in recovery. Parents provided information about their child’s condition and needs to enable the advocacy role of school-teachers and principals:

You know the principal called an assembly to inform the whole school about him and encouraging them not to laugh, instead be supportive when he comes back to school. That made the process much easier for him. [Parent FGD 2].

#### Acting against bullying

The child’s need to be protected from peer bullying was prominently reported on by both the children and their caregivers. Both parents and teachers at school would comfort or reassure the returning child around incidents of being laughed at, mocked or bullied, and offer practical advice and steps to ease the child’s return through e.g. minimizing obvious scars; while teachers and school principals would act against bullying by following up complaints, advocating to others on the child’s behalf, and educating the general school population and parents. In response to being laughed at in school, one child’s teacher educated the parents at the school on the consequences of the burn injury:

So he went again to school and on the third day his teacher called me concerned about him. She explained to me that all parents need to be called to a meeting where they can be educated about burns because the children were laughing at him every day. The teacher called all parents as she didn’t want to exclude certain children. So after that meeting my child was never bothered by other children at school. [Parent FGD 4].

## Discussion

This study highlights experiences of the supportive relationships that played a key role during hospitalization, the return home, and re-entry to school. Immediately after the trauma, it was the close family, supported by professional health staff, siblings and even initial visits by peers, that were key to providing physical presence, comfort and immediate affective support for the stabilization or psychological first aid of the child [32]. The return home, while initially experienced with relief, brought to the fore to the child and its immediate social network, the challenge of recognizing and integrating any changes to appearance or functionality, as it has for adolescents [33] and older survivors [21]. The longer-term psychosocial recovery was in this study centred around the preparations for re-entry into the school and anticipated experiences with the school peer community, where adult intervention and the placement of safeguarding measures to counter bullying were reported as critical for the reassurance and protection of the child [34].

This study draws attention, in the immediate aftermath of the trauma, to the salience of the family’s physical presence and unconditional affective support. The study highlighted parental and family presence, acts of comfort and emotional support, the preparation of extended family, and instrumental support. This helped alleviate the child’s anxiety, facilitated a sense of control over the situation, and assisted the child to make initial meaning of the trauma experience, as also indicated in other studies [33, 35, 36]. Here, the conscientious care by hospital staff beyond their clinical expertise including pain management, emphasized the interpersonal sensitivity and engagement with the child, especially where family members were not present. The latter was not uncommon, with parental presence often restricted whether due to economic barriers, or occupational and family pressures. Hospital volunteers, particularly those who are themselves burn survivors, appeared to be important as a manifestation of successful survival and coping of their own trauma. The importance of such emotional responsiveness, support, and empathy with the child’s experience, especially by parents or trusted parental figures, but also professional health staff, and installation of hope by volunteer burn survivor staff, was reported in this study as containing the alarm, confusion and distress that accompanied the child’s pain. Family support has already been indicated as a leading determining factor of psychosocial adjustment for child burn survivors [4, 37, 38], with parents offering comfort and both practical and emotional support to the child in an unfamiliar environment and especially during medical procedures [35]. Peer and sibling support has also been recognized as important in the recovery of the older child and adolescent, for whom acceptance by the peer group has been described as critical for psychosocial development [37]. In the hospital phase, it was the instrumental and emotional support and affection offered primarily by siblings, but also those that had been regarded as best friends and in a number of cases from friends made in hospital. The significance of such supportive relationships has also been echoed in child [39, 40], adult burn [41], and also other trauma recovery and resilience studies [42, 43]. In this study, the affection and support from family and friends, and the respect and compassion of hospital staff reassured and comforted the children, installed hope, and thus bolstered their endurance especially of the physical pain and helplessness highlighted immediately after the injury [19, 35], a form of psychological first aid [32].

Despite the initial relief after discharge a new set of challenges emerged and manifest when back at home, especially awareness of permanent physical, functional and body image changes, and the implications of these for social identity and self-esteem [19]. The removal of the dressings and over time recognition of the permanence of scars was reported here, as elsewhere [11, 37], to confront the child with the changes to their appearance that may initially have been ignored, and the feared implications of these for the child’s identity, esteem, and social relationships. This study highlighted that the recognition of the changes to appearance; the mobilization by the child of cognitive strategies to support post-burn social and daily activities; and the sharing of activities that had been enjoyed before, all contributed to the child’s post-burn adjustment. The appearance changes suffered have been reported elsewhere within a struggle for self-acceptance centered around the stark contrast of the ‘inner self’ with that of the now disfigured outer appearance, with the child needing to recognize that despite the external changes, their unique identity and inner self had or could endure and retain its value [33, 44]. The use of cognitive skills is aligned with indications that higher cognitive abilities are used to understand the burn incident, minimize negative self-attributions, and prepare for treatment and recovery experiences, all of which precede better social adjustment, coping and faster healing [12, 15]. The return to previous ‘normal’ interpersonal and recreational activities, especially when in public places and with neighborhood and later on school peers, has been reported to counter social isolation [37], reestablish with support a social presence and social competence, and bolster self-esteem [12]. This study indicates that such social and interpersonal engagements are necessary, especially when accompanied with publicly affirming experiences, along with positive cognitive strategies, to support an acceptance of the physical changes that had occurred. The latter psychological process has been identified as key to the child’s coping with the consequences of the trauma and the personal transformation required for recovery [12, 33], and preceded by the child’s positive reframing of the trauma and its consequences to “reconstruct their idea of themselves, their normality and their future” [22].

The child’s longer-term psychosocial recovery was in this study, as in others, reported as largely dependent on the survivor’s positive, supportive interactions with others, with these having promoted an easier reintegration first into their homes, and thereafter into their neighborhood, school and community at large [4, 11]. This study highlighted the importance of protective peer relationships to ease the re-entry of the child to school, buffer against the interpersonal challenges that would face the returning child, and support the child’s participation in school activities, including those such as sport, where the child’s visible injuries and scarring may be exposed and serve as a focus for stigmatization and bullying (see e.g. 4). This study thus recognized the school as an important setting for the returning child’s reengaging with peers, especially now in the absence of the previous direct support of hospital staff or parents, and as required for the formation and consolidation of peer and intimate relationships, the further development of the child’s self-image and social identity, and their academic competencies [45, 46]. While protective peer relationships with school peers was recognized, the reassurances, advice, corrective actions and proactive protection by educational authorities was also highlighted, along with the attendant need by such advocates for information on the child’s health and support needs, to ensure effective and supportive ways to foster the child’s school re-entry, even in the adverse school environments common in South Africa [46, 47]. The importance of schools designating an adult to which burn-surviving children can report bullying [48] is aligned to the role of adults highlighted here, while other disability studies have indicated a preference for support from peers above parental and teachers [49]. This advocacy role combined with the receptivity of school staff and peers to the returning child served to buffer against school re-entry concerns, and complemented the school reintegration enablers reported elsewhere, including the child’s emotional and interpersonal capacities and ongoing home support [50].

This study has a number of implications for the strengthening of practice directed at the recovery of burn injured children. Burn care facilities can, and in many cases do enable an emotionally supportive environment, involving wherever possible the family and the child in the management of treatment and rehabilitation, and facilitating the physical presence of families during hospitalization. Burn care in South Africa as in other under resourced settings, is however variable in terms of facilities and services, organization, staffing and workload. These facilities are furthermore predominantly emergency-driven and thus, along with South African health facilities in general, have been criticized for not sufficiently integrating the psychological, spiritual and social integration and recovery domains into physical resuscitation and rehabilitation plans [51]. This affects both in-hospital and post-discharge support, although the situation for post-discharge support is likely to be more precarious in under-resourced settings. This study highlighted strengthened post-discharge interpersonal support, and for this to include the child’s need for support to emotionally integrate the changes to appearance or functionality, support for the consolidation of the child’s post trauma identity, and for the positive reintegration into the neighborhood and in the school. However, this again must be considered within the context of generally overburdened health and social systems, where the affected family is expected to resume control over their own lives often with minimal input from specialized support staff, from the social and health [11], and educational systems [50].

### Strengths and limitations

This study is one of the first qualitative studies on early burn recovery and enablers, from the perspectives of the child, parent figures and staff [22], a combination which has been motivated for in child resilience research [27]. There is a contextualization of the known barriers to recovery, specific to the South African experience, but with an important emphasis on the enablers to recovery. These enablers were identified through perspectives offered from a range of people who have very close experience with the process. The complexities of the recovery experience were explored by an experienced research team with previous experience of qualitative and in-depth interviews with burn survivors. It is important to note that the interview guide was organized in a chronological manner so as to follow the process from hospital discharge to community reintegration, but also allowing for the authors’ emerging thoughts and reflections. The orientation, interview and debriefing conditions involved the detailed description of the purpose of study which was well understood from participants and contributed to a comfortable situation and environment. These interviews were in the home language of participants, to promote comfort, especially for the children, and to avoid misinterpretation.

Yet the study has a number of limitations. There may be some uncertainty as regards whose voice and what counts ‘most’ as enablers, as there were a number of enablers identified but how relatively important they are is not reflected in the material. There may also be an unequal representation of the situations that were meant to be covered, i.e. from the hospital time up to school reintegration, with the study e.g. not involving informants from all perspectives from the school. In addition, we know about school time from ‘projections’ and indirect accounts rather than from school peers and teachers themselves, and thus perhaps not all themes from this phase may be well covered. Also, we interacted with participants, in particular children, who were cared for in a highly regarded, specialized hospital setting in the Western Cape, and one of the few in the country with extensive experience in both the physical management and more importantly the psychosocial support of burn survivors that is aligned to the scientific literature on trauma recovery process [52]. This however, may have obscured concerns that may be more prevalent in other hospitals that have less specialized capacity.

## Conclusion

The study highlighted relational resources as key enablers of a child’s recovery after burns. Sensitive affective support and physical presence of key relationships, especially early on in the process, are crucial. The support for acceptance by the child and others of the visible changes in appearance causing issues with self-identity and self-esteem were emphasized, especially on the return home, as was the advocacy and protection required for the child’s re-entry back into the school and community.

## Supplementary information

**Additional file 1:.** Semi-Structured Interview Guide with Professionals

**Additional file 2:.** Child and Adolescent Focus Group Discussion Interview Schedule

**Additional file 3:.** Parent and Legal Guardian Focus Group Discussion Interview Schedule

## Data Availability

This is a qualitative study mirroring the context of the Western Cape, South Africa with a very small population of pediatric burn survivors, their caregivers and burn care specialists. Making the full data set publicly available could potentially be a breach to the privacy that the participants were promised upon request for participation. Also, our ethics approval from the South African Medical Research Council and the University of Cape Town’s Faculty of Health Sciences Ethics Committees was granted based on the anonymity of the individuals consenting to participate. Due to these conditions, the authors are unable to make the full transcripts available to a wider audience. Excerpts of specific segments of the text will be reviewed for any potentially identifying details and made available to fellow researchers or reviewers who complete a data sharing agreement and abide by strict confidentiality protocols. In line with the information given to the participants and restrictions set by the two ethical committees, access to the full transcripts are only available to the involved researchers. Data requests may be sent to the corresponding author, AVN, at ashley.vanniekerk@mrc.ac.za.

## Abbreviations

NGO: Non-governmental organization
FGD: Focus group discussion.

## Footnotes

1 1 Psychosocial refers to the mental, social, emotional and spiritual health of an individual with a typically complex interaction between these dimensions required for overall psychosocial well-being (59).
