# Supplementary material for: Enablers of psychosocial recovery in pediatric burns: perspectives from the children, parents and burn recovery support staff
Source: BMC Pediatr. 2020 Jun 9;20:289. doi: 10.1186/s12887-020-02180-z (PMC7282055; doi:10.1186/s12887-020-02180-z)
Supplement: Supplementary file 1 — Additional file 1:. Semi-Structured Interview Guide with Professionals [file 12887_2020_2180_MOESM1_ESM.docx]

# Appendix 1: Semi-Structured Interview Guide with Professionals

Person Interviewed: _____________________

Date: ___________________

Time: (start)______________(end)___________

Place: ________________________

Interviewed by: ___________________________

Duration: __________________

**Introduction:** Good morning/afternoon and thank you for agreeing to meet with us. The purpose of the study is to identify needs related to psychosocial recovery in pediatric burn victims. As someone who works with child and adolescent burn victims, we would like to ask you some questions about the types of psychosocial needs children and adolescents with burn injuries usually reported and what you observe in the course of your contact with them. Thank you for sharing your time with me and the interview should last not last more than 45 to 60 minutes. We will provide you with a summary of our results per email at the end of the study.

1. Can you tell me about the work you do? (Nature of the work; work with both children/adolescents and/or parents? Years of experience in the field; qualifications)
2. At what point in time do you first interact with a child or adolescent after a burn injury?
3. In your opinion what are the psychosocial issues faced by children and adolescents after a burn injury?

(Probe for blaming, avoidance, withdrawal, fear, not sleeping at night, not sleeping well, mood disruptions).

1. In your opinion what support needs do you observe that may not be reported by the children and adolescents or parents?
2. Could you share one or two stories of children and adolescents you worked with that had remarkable resiliency skills, ability to cope in the recovery phase. (Probe for skills or abilities that would be important for all children and adolescents to have in order to recover as quickly as possible with regards to psychosocial recovery).
3. Could you share one or two stories about what parents/caregivers, siblings and friends do to help a child recover better (Probe: how could protective factors be promoted in the child themselves and others around them? Give concrete examples of the kind of support you provide or that you know children and adolescents receive)
4. Based on your experience, what one to three messages do you think every child or adolescent should receive to assist in his/her psychosocial recovery?
5. Are there other issues or themes you would like to bring up that we have not discussed?

Thank you again for your assistance in better understanding the recovery needs and context of paediatric burn victims. The information generated from these questions will ultimately inform the development of a documentary that will provide psychosocial support to and strengthen the recovery process of young burn victims after they have been discharged from hospital.

**NB**: Ask if they could recommend anyone else for us to interview.
