# Supplementary material for: Enablers of psychosocial recovery in pediatric burns: perspectives from the children, parents and burn recovery support staff
Source: BMC Pediatr. 2020 Jun 9;20:289. doi: 10.1186/s12887-020-02180-z (PMC7282055; doi:10.1186/s12887-020-02180-z)
Supplement: Supplementary file 2 — Additional file 2:. Child and Adolescent Focus Group Discussion Interview Schedule [file 12887_2020_2180_MOESM2_ESM.docx]

# Appendix 2: Child and Adolescent Focus Group Discussion Interview Schedule

**Other**

**ME**

**Burn unit**

**Friends**

**Family**

**School**

| **Description** | **Time** |
| --- | --- |
| 1. **WELCOME**   Welcome and facilitators introduce themselves. | 2 min |
| 1. **OVERVIEW: Distribution and signing of Informed consent forms** 2. Reiterate the purpose of the discussion 3. Go through the assent form 4. Questions from participants 5. Signing of forms | 10 min |
| 1. **GROUND RULES**   Facilitate participants to formulate ground rules for the session. Participants will be given the opportunity to populate their own ground rules, however these below will be added to their list and visually be presented on newsprint in the room to serve as reminders:   1. No right or wrong answers, only differing points of view 2. It’s okay to skip questions; 3. There’s no right or wrong answers, 4. It’s okay to take a break or stop at any time 5. Confidential: What is shared in the group should be kept confidential by all participants (i.e. not sharing other people’s personal information to anyone outside of the group) 6. Respect: Don’t need to agree with others but should listen respectfully as others share their views and experiences 7. One person to speak at a time (recording) 8. Switch off cell phones | 5 min |
| 1. **ACTIVITY 1:** Icebreaker linked to participant introduction   **“Fly with me exercise”**  Provide all participants with an A4 sheet of paper and a pen. Show the group how to make a paper aeroplane. Then, allow each participant to make a paper aeroplane and write one thing they like and dislike on it. When everyone is ready instruct the group to fly their paper planes when you say “Go”, picking up a different plane every time. Repeat “Go” at least twice. After a minute or two, tell everyone to stop and pick up a paper plane and find the participant whose name is written on the paper and introduce this person (including their mentioned like and dislike) to the group. | 10 min |
| 1. **GROUP DISCUSSION:**   **GROUP DISCUSSION ACTIVITY: The description below is directed at question 1-5.**  Introducing participants to process: This flower has 5 petals (4 are burn unit, family, friends, school and the blank one is for you to decide, each representing an area in your life, with you at the centre. Today, we would like to discuss each of these areas (point children and adolescents to each of the levels/petals on the wall), to identify what can be said or done to help others of the same age recover better or faster. Here we want to focus on talking about the recovery process beyond the physical recovery. This includes how you felt, your emotions, thoughts, fears, reactions and behaviour.  **Let’s talk about:**   - 1. **The experiences of a child or adolescent at the burn unit and when going back home for the first time (i.e. preparing to leave the burn unit)**   **[Refer to petal “Burn Unit” on the wall].**   1. What can be said or done to help prepare a child or adolescent leaving the burn unit? (Probe for individual/internal as well as social/external support).  - What were the things you did to help yourself feel better? - What were the things others did to help you feel better?   1. **Being back home with family and siblings**   **[Refer to petal family on the wall.]**   1. What can be said or done to help a child or adolescent cope in the *first few days/weeks* home with family?  - What were the things *you* did to help yourself feel better? - What were the things *others* did to help you feel better?   1. **Re-connecting with friends**   **[Refer to petal friends on the wall]**   - - 1. What can be said or done to help a child or adolescent re-connect with their friends? - What were the things *you* did to help yourself feel better? - What were the things *others* did to help you feel better?   1. **Returning to school**   **[Refer to petal school on the wall]**   - - 1. What can be said or done to help a child or adolescent going back to school? - Prepare for going back to school? - Being at and dealing/managing/coping at school? - What were the things *you* did to help yourself feel better? - What were the things *others* did to help you feel better?   1. **Recovery process in general**   **[Refer to blank petal on the wall]** Now we have covered these general areas. Is there anything else you want to share that you think could help a child or adolescent recover emotionally or psychologically? | 60 min  (2)  (12)  (12)  (12)  (12)  (10) |
| SUMMARISE AND CONFIRMATION  Summarise main points and check understanding of what was shared. | 10 min |
| REVIEW  Review the purpose of the discussion and ask if anything has been missed. Mention that if anyone wants to talk, they can meet with us afterwards. | 5 min |
| Feedback questionnaire | 5 min |
| THANKS AND CLOSE SESSION |  |
|  | **95-107 min** |
