# Supplementary material for: Enablers of psychosocial recovery in pediatric burns: perspectives from the children, parents and burn recovery support staff
Source: BMC Pediatr. 2020 Jun 9;20:289. doi: 10.1186/s12887-020-02180-z (PMC7282055; doi:10.1186/s12887-020-02180-z)
Supplement: Supplementary file 3 — Additional file 3:. Parent and Legal Guardian Focus Group Discussion Interview Schedule [file 12887_2020_2180_MOESM3_ESM.docx]

# Appendix 3: Parent and Legal Guardian Focus Group Discussion Interview Schedule

**Other**

**My child**

**Burn unit**

**Friends**

**Family**

**School**

| **Description** | **Time** |
| --- | --- |
| 1. **WELCOME**   Welcome and facilitators introduce themselves. | 2 min |
| 1. **OVERVIEW: Distribution and signing of Informed consent forms** 2. Reiterate the purpose of the discussion 3. Go through the consent form 4. Questions from participants 5. Signing of forms | 10 min |
| 1. **GROUND RULES**   Facilitate participants to formulate ground rules for the session. Guidelines:   1. No right or wrong answers, only differing points of view 2. Confidential: What is shared in the group should be kept confidential by all participants (i.e. not sharing other people’s personal information to anyone outside of the group) 3. Respect: Don’t need to agree with others but should listen respectfully as others share their views and experiences 4. One person to speak at a time (recording) 5. Switch off cell phones | 5 min |
| 1. **ACTIVITY 1:** Icebreaker linked to participant introduction   **“Name Game”**   - One person starts by using an adjective starting with the same letter as their first name, followed by their first name, for example Sweet Silvia, Handsome Hank. - The next person repeats the adjective and first name of the first person, and then adds their own.   Continue around the circle, with the last person having to repeat all the names in order and adding their own. | 10 min |
| 1. **GROUP DISCUSSION:**   **GROUP DISCUSSION ACTIVITY: The description below is directed at question 1-5.**  Introducing participants to process: This flower has 5 petals (4 are burn unit, family, friends, school and the blank one is for you to decide, each representing an area in your child’s life with your child as the centre. Today, we would like to discuss each of these areas (point adults to each of the levels/petals on the wall), to identify what can be said or done to help children and adolescents recover better or faster. Here we want to focus on talking about the recovery process beyond the physical recovery. This includes how your children and adolescents felt, their emotions, thoughts, fears, reactions and behaviour.  **NB: Parents may want to emphasise their own experiences but ensure that the discussion remains focused on the child.**  **Let’s talk about:**   - 1. **The experiences of a child at the burn unit and when going back home for the first time (i.e. preparing to leave the burn unit)**   **[Refer to petal “Burn Unit” on the wall].**   1. What can be said or done to help prepare a child or adolescent leaving the burn unit? (Probe for individual/internal as well as social/external support).  - What were the things you did to help him/her feel better? - What were the things others did to help your child feel better?   1. **Being back home with family and siblings**   **[Refer to petal “Family” on the wall.]**   1. What can be said or done to help a child or adolescent cope in the *first few days/weeks* home with family?  - What were the things *you* did to help him/her feel better? - What were the things *others* did to help him/her feel better?   1. **Re-connecting with friends**   **[Refer to petal “Friends” on the wall]**   - - 1. What can be said or done to help a child or adolescent re-connect with their friends? - What were the things *you* did to help yourself feel better? - What were the things *others* did to help your child feel better?   1. **Returning to school**   **[Refer to petal “School” on the wall]**   - - 1. What can be said or done to help a child or adolescent going back to school? - Prepare for going back to school? - Being at and dealing/managing/coping at school? - What were the things *you* did to help yourself feel better? - What were the things *others* did to help your child feel better?   1. **Recovery process in general**   **[Refer to blank petal on the wall]** Now we have covered these general areas. Is there anything else you want to share that you think could help a child or adolescent recover emotionally or psychologically? | 60 min  (2)  (12)  (12)  (12)  (12)  (10) |
| SUMMARISE AND CONFIRMATION  Summarise main points and check understanding of what was shared. | 10 min |
| REVIEW  Review the purpose of the discussion and ask if anything has been missed. Mention that if anyone wants to talk, they can meet with us afterwards. | 5 min |
| THANKS AND CLOSE SESSION |  |
|  | **90-102 min** |
